# Relationships between Sleep Behaviors and Unintentional Injury in Southern Chinese School-Aged Children: A Population-Based Study

**DOI:** 10.3390/ijerph121012999

**Published:** 2015-10-16

**Authors:** Yafei Tan, Di Ma, Ying Chen, Fuyuan Cheng, Xiangxiang Liu, Liping Li

**Affiliations:** 1 Center for Injury Prevention Research, Shantou University Medical College, Shantou 515041, China; E-Mails: tanyf1012@163.com (Y.T.); 13chenying@stu.edu.cn (Y.C.); cfyhcf@163.com (F.C.); xxliu2014@126.com (X.L.); 2 Joint Shantou International Eye Center of Shantou University and Chinese University of Hong Kong, Shantou 515041, China; E-Mail: md@jsiec.org

**Keywords:** sleep patterns, sleep quality, unintentional injuries, Chinese school-aged children

## Abstract

The purpose of this study is to explore the relationships between sleep behaviors and injury occurrence among Chinese school-aged children. Data were collected with self-administered questionnaires of a cross-sectional survey which covered the school-aged children from southeastern Chinese urban and rural areas in April 2010. Information was collected on unintentional injury in the past year, sleep duration, napping and daytime fatigue, sleeping pill use, and social-demographic variables. Multivariable logistic regression analyses, controlling for confounding factors, were conducted to assess sleep-related variables that were associated with injuries. Students who slept for less than 8 h had a 30% increased risk of injury (OR: 1.30; 95%CI: 1.01–1.69) compared with those who slept for 8–9 h. Lack of napping, snoring and use of sleeping pills were significantly associated with injury. Among different genders, the slight difference in sleep behaviors predicted the occurrence of injury. Rural children displayed more sleep behaviors associated with injury than urban children. The sleep behaviors of primary school students were more negatively correlated with injury occurrence than junior/senior high school children. Consideration should be given to the prevention of problematic sleep behaviors as a potential risk factor in order to decrease injury rates and promote the health of school-aged children.

## 1. Introduction

Injury is a major cause of child and adolescent mortality in most countries, and is the leading cause of death for children 10–19 years of age [1,2,3]. Worldwide, around 830,000 children annually die from injuries, nearly 2300 each day [4]. In China, injury has become the first cause of death among 0–14-year-old. Nearly 50,000 child deaths are caused from injuries every year [5]. Hence, it is necessary to explore the causes of injury to prevent injury occurrence.

Sleep problems or poor sleep behaviors have been identified as risk factors for injury because they may impair motor function, mood, and cognitive functions through sleepiness, fatigue, and cognitive impairment [6,7]. Recent studies suggest that individuals with more poor sleep behaviors have significantly higher injury rates and more injury-prone behavior [8,9]. Several aspects of sleep behaviors may be hypothesized in relation to injury. For example, in terms of the relation between sleep duration and injury, several studies report that inadequate sleep hours and excessive sleep are both significantly associated with injury [10,11]. Inadequate sleep hours lead to daytime fatigue and sleepiness which compromise daytime performance through slow information processing, delayed response, increased reaction time, and reduced attentiveness. This may lead to negative social, behavioral, and health related outcomes [12] and can increase the risk for injury [13]. An Italian study found that inadequate sleep hours and lack of napping are related to an increased risk of injury in children [14], and other studies report that sleeping for less than 7.5 h per night increases the risk of injury by 61 percent compared with persons who sleep longer [15,16]. Recent studies show that not only sleep deprivation has an association with the occurrence of injury, but also excessive sleep poses a risk for injury as excessive sleep could be an indicator of poor physical and mental health status [15,17]. In contrast, another study also has shown that there is no direct association between sleep duration and injury [18]. Snoring has been defined as a risk factor associated with injury because snoring influences breathing and, in some cases even, sleep is repeatedly interrupted by apnea. Thus, snoring may result in poor quality of sleep and increase the risk of injury [19]. In addition, though sleeping at noon or napping is an individual behavior and it is not a culturally prescribed, normative behavior in China, students are encouraged to take a nap at noon because napping may repair mental, motor function, mood and cognitive functions and fatigue from morning courses, and lack of napping has proven to be a potential risk factor for injury among adolescents [20].

Adequate and high quality sleep have proven to be beneficial to the mental, physical and cognitive growth of children [7], but both medically- and behaviorally-based sleep problems are very common among children and are damaging to health [21,22,23]. Recent studies have reported that poor sleep quality among children 10–19 years of age is an independent predictor of health and performance outcome, including hypertension [24], metabolic diseases and mood, neurobehavioral problems such as hyperactivity and attention deficit [25,26], and academic achievement impairment [27]. These psychological and behavioral consequences may further contribute to increasing the risk of injury among children. Relative studies also report that injury rates are associated with sleep duration among 6–17-year-old children [16,28,29]. Owens and Li’s studies report a correlation among accidental injuries, increased injury risk behaviors, and sleep problems in children [28,30]. However, similar empirical evidence remains sparse for Chinese children. Because injury is a significant threat to child health, and poor sleep behaviors are associated with increasing risk of injury, examining the association of sleep behaviors with risk for injury among Chinese school-aged children is important.

Among 6–17-year-old Chinese school children or adolescents, there are only two studies that evaluated the possibility of a relationship between sleep duration and unintentional injury risks [16,28]. Both studies are effective in exploring the relationships between sleep duration and injury, finding that short sleep duration is significantly associated with injuries. However, the associations between other sleep-related variables and injury were not evaluated, and the samples were from a small, rural or urban area. Therefore, more comprehensive research that includes the elementary school to high school students and different sleep behaviors is needed to explore the relationships between sleep behaviors and elevated injury rate among Chinese school-aged children. We conducted this study to explore the potentially important and modifiable relationships between sleep behaviors and injury in southern Chinese school-aged children from elementary school to senior high school in different residential areas. We hypothesized that poor sleep behaviors would be highly associated with increased risks of injury among Chinese children. We further hypothesized that there were different sleep behaviors as a risk factor for injury among children of different gender, residential areas and school grades.

## 2. Methods

### 2.1. Study Population

A stratified cluster random-sampling method was applied to randomly select participants for this cross-sectional study, which was conducted in April 2010 in Shantou, China. All subjects were randomly enrolled from eight schools from elementary through high school. These randomly selected schools were comprised of three categories: four primary schools (two urban and two rural), two junior high schools (one urban and one rural) and two senior high schools (one urban and one rural), and four classes were randomly selected in every school grade. Considering that students in grades 1–2 were hard to understand and had difficulty answering the questionnaires independently, we only recruited pupils from grades 3–6 in the primary schools. All available students within the grades 3–12 from different schools were fully informed of the purpose of the investigation and participated voluntarily. All participants and their parents or guardians approved of the study and voluntarily signed written consent letters prior to the investigation. This study was approved by the Ethics Committee of the Medical College of Shantou University.

The self-report questionnaires were completed in the classroom within 20 min by voluntary individuals with a rigorously anonymous method. After being finished, all questionnaires were taken back by our professional investigators. In all, a total of 5435 students were invited to participate in the survey, and 4980 students fully completed the questionnaires, resulting in a 91.6% response rate.

It is necessary to emphasize that the questionnaire was self-designed. In order to test the reliability of the questionnaire used in the research, we took a pre-test among 50 students, and we retested the questionnaire 15 days later among the same individuals. The results showed that the questionnaire demonstrated good test-retest reliability with a correlation coefficient ranging from 0.813–1.000 (*p* < 0.000). The exploratory factor analysis was used to evaluate construct validity. The 10 common factors’ eigenvalues were more than one and the cumulative contribution rate was 69.5%. So, we deemed that the questionnaire had good reliability and validity, and the test-retest results showed the questionnaire design was scientific and reasonable.

### 2.2. Questionnaires and Measurements

A self-designed questionnaire was divided into four sections to collect participant information for this study, which contained demography and background information, behavioral and health outcomes and sleep-wake habits. In the first section, demography and background information were covered, such as age, grades, gender, residence type and family factors involving family economic status, parent jobs and parent education status.

Behavioral and health outcomes were examined in Section 2 and Section 3. Questions in those two sections involved: injury information that occurred in the past year, what they were doing when they suffered the injury, and the frequency at which the students smoked or drank. Other psychological conditions were assessed, such as have you felt depressed or hopeless in the past two weeks and do you frequently feel anxious, nervous, or worried? In our study, injury was restricted to unintentional injury, which was caused by something or someone without purpose and consciousness, defined as an accident that restricted normal activities for at least four hours or required medical attention, caused loss of consciousness, loss of awareness, or loss of memory for any length of time [31].

Section 4 contained 10 items concerning sleep-wake habits. The items of our sleep-wake habits questionnaires were based on a Chinese version of the Pittsburgh Sleep Quality Index (PSQI), which was validated to study child and adult sleep disturbance [32,33]. In order to know about the students’ sleep quality and sleep-wake habits, the items that were highly relevant for the purpose of the current study included: hours of sleep on average during the past month (including napping time), whether one took a nap after lunch, had trouble falling asleep, or difficulty falling asleep after waking up in the early morning, feeling tired when waking up in the morning, snoring when you are sleeping, frequencies of snoring per week, taking sleeping pills no less than once per week, feeling fatigue in the morning, and having trouble staying awake during the day. For analysis, according to the recommended sleep hours of students from Chinese Ministry of Education, sleep duration was categorized into three levels: less than 8, 8–9, and more than 9 h. Response categories for some questions, such as frequency of snoring or fatigue, were separated into three categories: (1) never/rarely or a few nights/days per month; (2) once or several nights/days per week; (3) every night or every day.

### 2.3. Statistical Analysis

Descriptive statistics were generated to evaluate univariate relationships with injuries as the outcome variable, including total and injury frequencies and percentages in different variables. Chi-square tests were used to compare frequencies on different levels of categorical socio-demographic factors. Multiple regression analysis was used to determine associations between sleep-related variables and injury by controlling gender, family income, father’s educational level, residence type, school grades (age was excluded for collinearity with school grades), physical activities, smoking, alcohol drinking and depression anxiety and status. The interactions of sleep behaviors with gender, residence types and school grades to injury were examined. The interactions were evaluated by hierarchical multiple regression analysis calculating the adjusted ORs of injury for sleep-related variables separately in different genders, residence types and school grades. All analyses were conducted using SPSS version 21.0 software (SPSS Inc., Chicago, IL, USA). Significance was assessed based on a two-tailed test with a critical value set at 0.05.

## 3. Results

Among the 4980 students in the sample, including 52.9% boys and 47.1% girls, 2796 (56.1%) students were from rural schools and 2184 (43.9%) students were recruited form urban schools. The average age was 12.7, with a range of 9–18 years. During the past year, a total of 24.4% of students had an injury that restricted normal activities for at least four hours or required medical attention.

Table 1 shows the associations between social-demographic characteristics and injury. Concerning sleep behaviors, injury occurrence was associated significantly with all of the sleep variables. In terms of psychological status, depression and anxiety status were inclined to predict injury incidence for students.

**Table 1:** Social-demographic characteristics and injuries among school-aged students.

Variables		Total (%)	Injured		*p*-Value
			n	%	
Gender					<0.001
	Girls	2347 (47.1)	450	19.2	
	Boys	2633 (52.9)	765	29.1	
Grade					<0.001
	3–6	2763 (55.5)	762	27.6	
	7–9	1029 (20.7)	190	18.5	
	10–12	1188 (23.8)	263	22.1	
Residence type					<0.001
	Urban	2184 (43.9)	637	29.2	
	Rural	2796 (56.1)	578	20.7	
Parent marital status					0.100
	Divorced	280 (5.8)	80	28.6	
	Married	4534 (94.2)	1098	24.2	
Single-child family					0.648
	No	3767 (76.5)	907	24.1	
	Yes	1159 (23.5)	294	25.4	
Living in school					0.526
	No	4089 (84.8)	986	24.1	
	Yes	721 (15.2)	188	26.1	
Father’s educational level					0.007
	Primary school graduate or less	924 (22.0)	246	26.6	
	Junior/Senior high school graduate	2530 (60.2)	574	22.7	
	College/university graduate or above	747 (17.8)	210	28.1	
Mother’s educational level					0.064
	Less than primary school graduate	1900 (39.6)	428	22.5	
	Junior/Senior high school graduate	2267 (47.3)	567	25.0
	College/University graduate or above	627 (13.1)	171	27.3	
Total family income					0.001
	High	2117 (42.5)	514	24.3	
	Average	2237 (44.9)	513	22.9	
	Low	626 (12.6)	188	30.0	
Ever smoked					<0.001
	No	4077 (84.5)	920	22.6	
	Yes	745 (15.5)	258	34.6	
Alcohol consumption					<0.001
	No	2800 (58.4)	563	20.1	
	Yes	1994 (41.6)	615	30.8	
Physical activity per day (hours)					<0.001
	<0.5	3429 (71.1)	801	23.4	
	≥0.5 ≤1	944 (19.6)	234	24.8	
	>1	448 (09.3)	162	36.2	
Duration of sleep (hours)					<0.001
	<8	624 (12.5)	174	27.9	
	≥8, ≤9	2980 (59.8)	660	22.1	
	>9	1376 (27.7)	381	27.7	
Napping					<0.001
	No	2355 (47.3)	733	31.1	
	Yes	2625 (52.7)	482	18.4	
Trouble falling asleep					<0.001
	No	2944 (63.9)	635	21.6	
	Yes	1661 (36.1)	481	29.0	
Waking up in middle of night or early morning					<0.001
	No	3280 (74.8)	758	23.1	
	Yes	1105 (25.2)	314	28.4	
Snoring					<0.001
	No	3202 (64.3)	745	23.3	
	Yes	380 (7.6)	127	33.4	
	Unknown	1398 (28.1)	343	24.5	
	Never/Rarely or a few nights per month	3210 (64.5)	753	23.5	
	Once or several nights per week	147 (3.0)	58	39.5	
	Every night	66 (1.3)	27	40.9	
	Unknown	1557 (31.2)	377	24.2	
Feeling fatigue in the morning					0.001
	Never/Rarely or a few days per month	2702 (54.3)	624	23.1	
	Once or several days per week	823 (16.5)	203	24.7	
	Every day	556 (11.2)	174	31.3	
	Unknown	899 (18.1)	214	23.8	
Daytime fatigue					<0.001
	No	3831 (88.1)	902	23.5	
	Yes	516 (11.9)	164	31.8	
Sleeping pill use per week					<0.001
	No	4191 (97.9)	1009	24.1	
	Yes	89 (2.1)	37	41.6	
Depressed symptom					<0.001
	No	3965 (87.2)	916	23.1	
	Yes	580 (12.8)	204	35.2	
Anxiety					<0.001
	No	4242 (93.3)	1022	24.1	
	Yes	304 (6.7)	106	34.9	

Multivariable logistic regression analysis was performed to test the association of sleep behaviors with injury (Table 2). The regression model had a good fit, the log-likelihood value was 239.432, the nagelkerke R-squared value was 0.537 and the sensitivity and specificity of determining injury was 82.3% and 94.2%. Compared to sleeping for 8–9 h, sleeping for less than 8 h was significantly associated with an increase in injury (OR = 1.30, 95%CI = 1.01–1.69). Students who did not take a nap had a 1.92-fold higher risk of injury compared to students who took a nap (OR = 1.92, 95%CI = 1.63–2.26). Trouble falling asleep (OR = 1.35, 95%CI = 1.15–1.59), waking up in the middle of night (OR = 1.19, 95%CI = 1.00–1.42), snoring (OR = 1.44, 95%CI = 1.03–2.02), and using sleeping pills (OR = 2.05, 95%CI = 1.18–3.57) were significant in predicting the risk for injuries. However, daytime fatigue and snoring frequency were not significant in predicting the risk for injuries.

In order to determine whether the relationships between sleep behaviors and injury differed among boys and girls, the hierarchical multiple regression analysis was conducted (Table 3). In the first step of regression, the sleep-wake variables and confounding factors are entered into the model as predictors of the injury. The independent variables have to be significant predictors of the injury to test for an interaction in the next step. Then, separate regression equations may be required with each gender to explore the interaction with injury. The parent marital status as an instrument variable was included in the first stage of the regression equation. In the test of the regression model, the log-likelihood value was 339.432, the nagelkerke R-squared value was 0.493 and the sensitivity and specificity of determining injury was 85.7% and 95.2%. Significant risk factors associated with injury in both genders were lack of napping and trouble falling asleep. For sleeping pill use, boys had a greater risk for injury than girls (OR = 2.65, 95%CI = 1.27–5.52). In addition, in the snoring frequency once or several nights per week groups, girls had a higher risk for injury than boys (OR = 2.67, 95%CI = 1.27–5.61).

**Table 2:** Adjusted odds ratios for injury obtained from multivariable logistic regression analysis among children.

Variables		OR	95%CI	
Sleep duration (hours)				
	≥8, ≤9	1.00		
	<8	1.30 *	1.01	1.69
	>9	1.08	0.90	1.29
Feeling fatigue when waking up in the morning				
	Never/Rarely or a few days per month	1.00		
	Once or several days per week	0.99	0.80	1.25
	Every day	1.18	0.93	1.51
Napping				
	Yes	1.00		
	No	1.92 ^#^	1.63	2.26
Trouble falling asleep				
	No	1.00		
	Yes	1.35 ^#^	1.15	1.59
Waking up in the middle of the night or early morning				
	No	1.00		
	Yes	1.19 *	1.00	1.42
Snoring				
	No	1.00		
	Yes	1.44 *	1.03	2.02
Snoring frequency				
	Never/Rarely or a few nights per month	1.00		
	Once or several nights per week	1.07	0.68	1.69
	Every night	1.29	0.66	2.53
Daytime fatigue				
	No	1.00		
	Yes	1.20	0.95	1.53
Use of sleeping pills once or more per week				
	No	1.00		
	Yes	2.05 *	1.18	3.57

Notes: Adjusted for gender, school grades, family income, father’s educational level, residence type, smoking, alcohol use, physical activity, and depression and anxiety status. OR: odds ratio. 95%CI: 95% confidence interval. * *p* < 0.05; **^#^** *p* < 0.01.

**Table 3:** Adjusted ORs for injury obtained from hierarchical multiple logistic regression analysis in boys and girls.

Variables		Boys			Girls		
		OR	95%CI		OR	95%CI	
Sleep duration (hours)							
	≥8, ≤9	1.00			1.00		
	<8	1.17	0.83	1.64	1.42	0.91	2.22
	>9	0.99	0.78	1.26	1.08	0.82	1.42
Feeling fatigue when waking up in the morning							
	Never/Rarely or a few days per month	1.00			1.00		
	Once or several days per week	1.17	0.88	1.57	0.79	0.55	1.13
	Every day	1.23	0.90	1.69	1.18	0.79	1.76
Napping							
	Yes	1.00			1.00		
	No	1.82 ^#^	1.46	2.26	1.98 ^#^	1.53	2.56
Trouble falling asleep							
	No	1.00			1.00		
	Yes	1.30 *	1.05	1.60	1.39 *	1.07	1.80
Waking up in middle of night or early morning							
	No	1.00			1.00		
	Yes	1.18	0.94	1.49	1.22	0.92	1.62
Snoring							
	No	1.00			1.00		
	Yes	1.48	0.97	2.24	1.53	0.84	2.82
Snoring frequency							
	Never/rarely or a few nights per month	1.00			1.00		
	Once or several nights per week	0.75	0.42	1.36	2.67 *	1.27	5.61
	Every night	1.30	0.59	2.85	1.11	0.27	4.51
Daytime fatigue							
	No	1.00			1.00		
	Yes	1.06	0.77	1.45	1.41	0.96	2.08
Use of sleeping pills once or more per week							
	No	1.00			1.00		
	Yes	2.65 *	1.27	5.52	1.61	0.60	4.32

Notes: Adjusted for school grades, residence type, family income, father’s educational level, smoking, alcohol use, physical activity, and depression and anxiety status. OR: odds ratio. 95%CI: 95% confidence interval. * *p* < 0.05; **^#^** *p* < 0.01.

The association between injury and different sleep behaviors, as a function of residence type, was explored by classification of urban students and rural students using hierarchical multiple regression analysis (Table 4). The regression model had a good fit, the log-likelihood value was 215.376, the nagelkerke R-squared value was 0.637 and the sensitivity and specificity of determining injury was 89.3% and 97.3%. The living in school as an instrument variable was included in the first stage of the regression equation. Among the urban students, lack of napping (OR = 1.83, 95%CI = 1.41–2.37), and trouble falling asleep (OR = 1.43, 95%CI = 1.12–1.83) predicted the occurrence of injury, as well as some other sleep-related variables. Among the rural students, lack of napping (OR = 2.04, 95%CI = 1.64–2.55) and trouble falling asleep (OR = 1.34, 95%CI = 1.07–1.68) were also highly related to an increased risk of injury. In addition, the students from rural areas who snored (OR = 2.03, 95%CI = 1.26–3.27) and used sleeping pills (OR = 2.44, 95%CI = 1.18–5.05) were associated with occurrence of injury. The rural students who had sleep problems had a higher risk of injury than those urban students.

**Table 4:** Adjusted ORs for injury obtained from hierarchical multiple logistic regression analysis in urban and rural children.

Variables		Urban			Rural		
		OR	95%CI		OR	95%CI	
Sleep duration (hours)							
	≥8, ≥9	1.00			1.00		
	<8	1.28	0.85	1.87	1.40	0.95	2.05
	>9	0.85	0.65	1.11	1.16	0.91	1.48
Feeling fatigue when waking up in the morning							
	Never/Rarely or a few days per month	1.00			1.00		
	Once or several days per week	1.14	0.81	1.59	1.00	0.73	1.35
	Every day	1.11	0.75	1.64	1.28	0.92	1.79
Napping							
	Yes	1.00			1.00		
	No	1.83 ^#^	1.41	2.37	2.04 ^#^	1.64	2.55
Trouble falling asleep							
	No	1.00			1.00		
	Yes	1.43 *	1.12	1.83	1.34 *	1.07	1.68
Waking up in the middle of the night or early morning							
	No	1.00			1.00		
	Yes	1.14	0.86	1.52	1.19	0.94	1.51
Snoring							
	No	1.00			1.00		
	Yes	0.89	0.54	1.48	2.03 *	1.26	3.27
Snoring frequency							
	Never/Rarely or a few nights per month	1.00			1.00		
	Once or several nights per week	1.44	0.81	1.59	0.96	0.48	1.91
	Every night	2.00	0.75	1.64	0.68	0.26	1.75
Daytime fatigue							
	No	1.00			1.00		
	Yes	1.07	0.72	1.59	1.14	0.83	1.57
Use of sleeping pills once or more per week							
	No	1.00			1.00		
	Yes	1.77	0.66	4.74	2.44 *	1.18	5.05

Notes: Adjusted for gender, school grades, family income, father’s educational level, smoking, alcohol use, physical activity and depression and anxiety status. OR: odds ratio. 95%CI: 95% confidence interval. * *p* < 0.05; **^#^** *p* < 0.01.

To further examine the potential association between sleep behaviors and injury among different grade groups, hierarchical multiple regression analysis was conducted after controlling confounding factors (Table 5). In the regression model test, the log-likelihood value was 247.249, the nagelkerke R-squared value was 0.623 and the sensitivity and specificity of determining injury was 84.9% and 96.5%. The living in school as instrument variable was included in the first stage of regression equation. For primary school students and senior high school students, sleeping less than 8 h was highly associated with injury risks (OR = 1.88, 95%CI = 1.07–3.31; OR = 1.51, 95%CI = 1.04–2.18, separately), but there was no significant association for junior high school students. Lack of napping was also a risk factor for injury among primary (OR = 2.05, 95%CI = 1.67–2.52) and senior high school students (OR = 2.26, 95%CI = 1.41–3.62). The primary and senior high school students who did not have a habit of napping were more likely to suffer injury than junior high school students. In addition, primary and junior high school students who had trouble falling asleep had increased odds of an injury. Snoring was a risk factor for injury among primary students (OR = 1.71, 95%CI = 1.11–2.65). Using sleeping pills was a risk factor of injury among different grade groups, but there was no difference among primary and senior high school groups.

**Table 5:** Adjusted ORs for injury obtained from hierarchical multiple logistic regression analysis in different grades groups.

Variables		Primary School			Junior High School			Senior High School		
		OR	95%CI		OR	95%CI		OR	95%CI	
Sleep duration (hours)										
	≥8, ≤9	1.00			1.00			1.00		
	<8	1.88 *	1.07	3.31	0.55	0.23	1.33	1.51 *	1.04	2.18
	>9	1.02	0.82	1.26	1.05	0.68	1.63	1.33	0.65	2.75
Feeling fatigue when waking up in the morning										
	Never/Rarely or a few days per month	1.00			1.00			1.00		
	Once or several days per week	1.13	0.83	1.56	0.79	0.44	1.41	0.98	0.65	1.49
	Every day	1.29	0.91	1.83	0.75	0.41	1.36	1.43	0.86	2.38
Napping										
	Yes	1.00			1.00			1.00		
	No	2.05 ^#^	1.67	2.52	1.48	0.99	2.21	2.26 *	1.41	3.62
Trouble falling asleep										
	No	1.00			1.00			1.00		
	Yes	1.39 *	1.12	1.73	1.69 *	1.13	2.54	1.29	0.90	1.84
Waking up in middle of night or early morning										
	No	1.00			1.00			1.00		
	Yes	1.21	0.96	1.53	1.02	0.66	1.58	1.31	0.87	1.97
Snoring										
	No	1.00			1.00			1.00		
	Yes	1.71 *	1.11	2.65	0.80	0.32	2.03	1.15	0.52	2.52
Snoring frequency										
	Never/Rarely or a few nights per month	1.00			1.00			1.00		
	Once or several nights per week	0.84	0.48	1.49	1.56	0.40	6.09	2.76	0.88	8.65
	Every night	1.11	0.43	2.84	1.47	0.37	5.86	0.54	0.09	3.47
Daytime fatigue										
	No	1.00			1.00			1.00		
	Yes	1.31	0.92	1.86	1.15	0.64	2.06	0.84	0.54	1.32
Use of sleeping pills once or more per week										
	No	1.00			1.00			1.00		
	Yes	2.14	0.96	4.78	3.94 *	1.08	14.42	1.12	0.31	4.07

Notes: Adjusted for gender, residence type, family income, father’s educational level, smoking, alcohol use, physical activity and depression and anxiety status. OR: odds ratio. 95%CI: 95% confidence interval. * *p* < 0.05; **^#^** *p* < 0.01.

## 4. Discussion

This study focuses on the relationship between sleep behaviors and unintentional injury among school-aged children in China, and the results highlight specific aspects of sleep behaviors that are associated with increased likelihood of an injury, such as sleep duration, lack of napping, trouble falling asleep, snoring and sleeping pill use. Among the stratified groups, lack of napping and trouble falling asleep are consistent risk factors for injury among the different groups. Boys who used sleeping pills were more at risk for injury than that of girls, whereas girls who snored once or several nights per week were more at risk for injury than that of boys. Children from rural areas who snored and used sleeping pills had greater risk than urban children. Furthermore, grade-stratified analyses shows primary students who have poor sleep behaviors are more likely to suffer injury than high school students. To the best of our knowledge, this is one of the few comprehensive and large-scale cross-sectional studies which cover different residents, genders and school grades to evaluate the correlation between sleep behavior and injury among Chinese school-aged children.

Adequate sleep duration or good sleep quality are important for maintaining daytime alertness and can prevent the occurrence of injury [15]. Our results demonstrate that sleeping for less than 8 h, trouble falling asleep, waking up early and using sleeping pills which included prescription medication or over the counter pills are associated with increased rate of injury. Several studies support that sleeping less than 8 h and having trouble falling asleep lead to inadequate sleep, and children who wake up early and use sleeping pills are likely to have poor sleep quality and inadequate sleep, which would increase their risks of injury [31,34]. A possible explanation for the relationship is that poor sleep quality and inadequate sleep for long periods may lead to subjective and objective daytime sleepiness, which would result in concentration loss and carelessness, and further increase the risk of injury occurrence in children [7,35,36]. Lack of napping has been proven as a predictor of injury in children, which is consistent with a prior study [14], partly because napping can reduce the potential hours that one could get hurt. Besides, sleeping at noon may repair mental, motor function, mood and cognitive functions and fatigue from morning courses. Furthermore, snoring in children is associated with risk for injury in this study, which is supported by other studies showing that snoring or sleep-disordered breathing are important risk factors for injury [29,30]. However, we find that daytime fatigue is not associated with injury in this study. This is in contrast to other studies that found that daytime fatigue is a significant risk factor for injury [28,29]. One possible explanation is that children with fatigue in the daytime tend to sleep and relax quietly, thereby decreasing the risk of injury occurrence.

The role of gender in modifying the relationship between sleep behaviors and injury has been identified in this study. Boys who used sleeping pills have a higher risk for injury than that of girls, and girls who snore once or several nights per week have a higher risk for injury than that of boys, and there is no significant difference among boys and girls in other injury-associated sleep behaviors. The difference might be explained by the fact that boys are more energetic and active and are more likely to take injury risks than girls, and there are also different sleep needs among girls and boys [26,37]. Alternatively, the perception of sleepiness might be different between boys and girls, which could influence efforts to take preventive actions at different levels of sleepiness [38].

Though the incidence of injury among urban children is higher than rural children in this study, the rural children with poorer sleep behaviors have higher risks for injury than urban children when the analysis was residence stratified. Results suggest that poor sleep quality is the reason for injury in rural children. Stallones, with similar results in their study, showed that rural children had worse sleep quality and were more likely to injure themselves [31]. Huang *et al.* [39] found that there was a larger incidence of co-sleeping among rural children than urban children because of the differences in socio-economic status and social values, and the sleep behaviors of children were easily influenced by co-sleeping, which tended to be associated with later bedtimes, bedtime resistance and daytime sleepiness, thereby, increasing the probability of risk of injury among rural children. Furthermore, compared to urban areas, there are more potential risk factors for injury in rural areas, which further contributes to the occurrence of injury [40].

It has been estimated that young children with an increased prevalence of sleep disturbances have higher injury rates and more injury-prone behavior than older children [30,31]. The results of this study support the hypothesized relationship between poor sleep behavior and injury in Chinese school-aged children. We demonstrate that the pupils who have sleep problems are significantly more prone to injury risks than junior and senior high school children. Primary school children who sleep less than 8 h, lack of napping, trouble falling asleep and snoring have more risks associated with injury than older children. Young children who suffer sleep deprivation are vulnerable to injury because the children’s autonomous control consciousness has not yet been developed and they are easily influenced by their health status and living environment [30]. Further, behavioral and social changes may influence sleep behavior-related injury risks differently among older children compared to younger children [37]. In conclusion, different sleep needs and individual conditions contribute to different sleep behavior-related injury risk factors among different age groups. Therefore, adequate sleep hours and high sleep quality are a prerequisite for preventing injury in young children.

Several potential limitations should be considered. First, we did not classify the category of injuries, so the relationships between sleep behaviors and different injury types were not explored. Second, we restricted our target injuries to include only those that restricted normal activity for at least half a day or required medical attention in the past year. In doing so, we might have potentially excluded incidents involving slight injury in the study design and undermined the associations between sleep behaviors and injury. Third, the data was reported by children themselves, meaning that the results might have been influenced by recall bias and measurement error. Furthermore, self-report bias might lessen the accuracy of the results because the information might be susceptible to self-perception and interpretation, such as in the case of snoring, which is more likely to be prone to error from self-reporting [16]. Fourth, other factors, such as co-sleep, dangerous games or other high risk behaviors and children’s BMI, have not been taken into consideration in our study. Several previous studies showed that these factors could affect the sleep behaviors or play a positive/negative role in increasing risk of injury [39,40]. Finally, this was a cross-sectional study where we only summarized some tentative conclusions about the relationship between poor sleep behaviors and injury. Longitudinal research is needed to help us better understand the inter-relationships and underlying mechanisms.

## 5. Conclusions

There is an increased prevalence of sleep problems among Chinese school-aged children with increased injury rates, and specific aspects of sleep behaviors are associated with increased risk of injury. Further studies are desirable to confirm and strengthen our results by using prospective designs and including multiple variables of injuries and injury-prone behaviors.

From the perspective of promoting children’s health, our results highlight that prevention strategies, such as ensuring sufficient sleep durations or encouragement to take naps, which can improve sleep quality and reduce resulting injuries, should be formulated according to the different needs of each individual. Screening and identification of poor sleep behaviors or sleep problems among children, especially for young and rural children, may be an important step in injury prevention. We also suggest that consideration from parents and teachers should be given to young children to support their sleep quality and reduce poor sleep behaviors as a potential risk factor of increased injury rates.
